# Magnetic Resonance Neurography Within a Multimodal Framework for Predicting C5 Root Graftability in Traumatic Brachial Plexus Injury

**DOI:** 10.2106/JBJS.OA.26.00123

**Published:** 2026-06-09

**Authors:** Ying-Hsuan Lee, Yenpo Lin, Yu-Ching Lin, Tommy Nai-Jen Chang, David Chwei-Chin Chuang, Johnny Chuieng-Yi Lu

**Affiliations:** 1 Department of Plastic and Reconstructive Surgery, Chang Gung Memorial Hospital, Chang Gung University, Taoyuan, Taiwan; 2 Department of Radiology, Chang Gung Memorial Hospital, Chang Gung University, Taoyuan, Taiwan; 3 Division of Plastic and Reconstructive Surgery, Department of Surgery, Wan Fang Hospital, Taipei Medical University, Taipei, Taiwan; 4 Division of Plastic Surgery, Department of Surgery, Memorial Sloan Kettering Cancer Center, New York, New York

## Abstract

**Background:**

Accurate preoperative assessment of C5 nerve root graftability is crucial for surgical planning in brachial plexus injuries. This study evaluates the diagnostic accuracy of magnetic resonance neurography (MRN) compared with clinical examination and electrodiagnostic studies in predicting C5 graftability.

**Methods:**

This retrospective cohort study included 402 adult traumatic brachial plexus injuries patients undergoing nerve reconstruction at a single tertiary center between September 2008 and November 2024. Patients were categorized into panplexus (C5-T1, n = 166) and non-panplexus (n = 236) injuries. Diagnostic modalities included physical examination (deafferentation pain, Tinel’s sign, rhomboid, and levator scapulae muscle power [MP]), electromyography of C5 paraspinal and rhomboid muscles, and high-resolution MRN with avulsion severity scoring. Intraoperative surgical inspection served as the reference standard. Multivariate logistic regression and receiver operating characteristic curve analysis evaluated diagnostic performance.

**Results:**

Intraoperative exploration revealed graftable C5 roots in 195 patients (48.5%). MRN demonstrated the highest predictive value (odds ratio 9.171, p < 0.001). Optimal diagnostic models varied by injury pattern: Panplexus injuries showed optimal prediction with MRN plus Tinel’s sign (area under the receiver operating characteristic curve 0.828), while non-panplexus injuries required MRN, Tinel’s sign, and levator scapulae MP (area under the receiver operating characteristic curve 0.766). Electromyography showed limited diagnostic value.

**Conclusions:**

MRN is a valuable tool for assessing C5 nerve root graftability, demonstrating the highest predictive performance among evaluated modalities. When combined with clinical examination, it supports structured preoperative decision making in brachial plexus reconstruction.

**Level of Evidence:**

Diagnostic Level II. See Instructions for Authors for a complete description of levels of evidence.

## Introduction

Root availability in brachial plexus injuries (BPI) can decide reconstructive strategies for severe patterns of injuries, often challenged by the paucity of available donor nerves^1^. Central to this assessment is distinguishing between ruptured and avulsed spinal nerve roots, as this fundamentally determines reconstructive strategy^2^. In avulsion injuries, the root is torn from the spinal cord, rendering it nongraftable and necessitating alternative strategies such as nerve transfers. Conversely, ruptured spinal nerves allow nerve grafting, which provides additional source of donor nerves that can improve functional outcomes and spares donor nerves for other functions^3-6^. Among cervical nerve roots, C5-C6 more commonly sustain rupture while maintaining proximal continuity, making nerve grafting feasible^7^. Nevertheless, accurate C5 graftability prediction remains challenging. Current modalities include physical examination (i.e. deafferentation pain, Tinel’s sign, muscle power [MP] assessment), electromyography (EMG), and imaging^8-10^. No single test has proven sufficient, and variable accuracy often leads to conflicting results confounding surgical decision making^11^. This uncertainty is particularly problematic in panplexus injuries where extensive injury limits clinical examination reliability and donor nerve options are more constrained^12^.

Magnetic resonance neurography (MRN) has emerged as a promising diagnostic tool for evaluating nerve root integrity in BPI. Our institution has incorporated advanced high-resolution techniques to facilitate direct visualization of intradural ventral and dorsal rootlets^13,14^. The purpose of this study was to evaluate the diagnostic performance of MRN in predicting C5 nerve root graftability compared with clinical examination and electrodiagnostic studies and to develop optimal prediction models for different injury patterns. This multidisciplinary study presents MRN findings in 402 adult patients with BPI, the largest series to date detailing imaging findings to surgical exploration.

## Materials and Methods

### Study Design and Participants

This retrospective cohort study was conducted at Chang Gung Memorial Hospital from September 2008 to November 2024. Inclusion criteria were (1) traumatic BPI, (2) age18 years or older, and (3) preoperative MRN imaging per institutional protocol. Patients were stratified by injury pattern into panplexus injuries (C5-T1 root involvement) and non-panplexus injuries (C5-6, C5-7, or C5-8) depending on clinical examination, electrodiagnostic and imaging findings, and surgical exploration. Demographic data including gender, body mass index, age, and race/ethnicity were recorded. Race and ethnicity were obtained from medical records as part of routine patient registration (self-identified). This study was approved by the Institutional Review Board of Chang Gung Memorial Hospital (CMRPG3P0711).

## Preoperative Assessment Protocol

### Clinical Evaluation

All patients underwent comprehensive evaluation by experienced brachial plexus nerve surgeons (faculty with >10 years of peripheral nerve surgery experience) using standardized protocols including (1) documentation of deafferentation pain (severe burning neuropathic pain in the affected limb radiating from the neck down to the hand, without a dermatomal distribution); (2) muscle strength testing of C5-innervated muscles, rhomboid, and levator scapulae, using Medical Research Council scale M0-M5. Rhomboid strength was tested with the patient attempting to touch the shoulder blades together and observing closely for symmetry; levator scapulae by asking the patient to place their hand in their back pocket (internal rotation/adduction) and then shrug. This inhibits the trapezius and isolates the levator/rhomboids; (3) Tinel's sign elicited by percussion over the supraclavicular region at either the Erb’s point or the posterior cervical triangle. A positive test was defined as reproduction of paresthesia radiating into the C5 dermatome distribution.

### Electromyographic Assessment

EMG evaluated C5 paraspinal and rhomboid muscle function, providing information on dorsal scapular nerve integrity and C5 root viability. EMG findings were dichotomized as preserved (detectable voluntary motor unit recruitment) or denervated (absent or severely reduced recruitment).

### Magnetic Resonance Imaging Protocol

High-resolution MRN was performed on a 3T scanner (Discovery MR750w, GE Healthcare) using head, neck, and torso coils. The protocol comprised 3 sequences: (1) fast imaging employing steady-state acquisition (FIESTA) neurography (0.6-0.8 mm^3^ resolution) for intradural root visualization; (2) Short TI inversion recovery for extraforaminal rupture assessment; and (3) diffusion-weighted imaging for dorsal root ganglion evaluation. Images were acquired by dedicated technologists and interpreted by 2 experienced peripheral nerve radiologists using a Picture Archiving and Communication System workstation (Centricity Universal Viewer; GE Healthcare). All MRN studies were performed at least 2 months after injury to optimize visualization of the nerve roots and minimize the effects of acute tissue edema.

### MRN-Based Root Avulsion Severity Classification

We applied a standardized avulsion severity scoring system based on coronal reformatted FIESTA MRN, previously developed and validated at our institution^15^. Normal C5 intradural anatomy consists of 5 symmetric ventral motor and dorsal sensory rootlets comparable to the contralateral side. Avulsion severity was graded as normal (N), partial avulsion (P1-P4, reflecting loss of 1-4 rootlets), or complete avulsion (C). Both motor and sensory rootlets were assessed, as preservation of both is critical for functional recovery. Roots graded N or P1-P2 were considered graftable, whereas P3-P4 and C roots were deemed nongraftable.

### Reference Standard

C5 graftability was determined by compatibility between MRN evaluation and surgical inspection. Direct surgical inspection was performed by 2 brachial plexus surgeons with more than 10 years of experience. To minimize bias, graftability was confirmed through a strict hierarchy of evidence: (1) visual identification of healthy fascicular “mushrooming” under magnification; (2) positive rhomboid motor response to dorsal scapular nerve stimulation; and (3) absence of a pseudomeningocele from C5. Only spinal nerves meeting these criteria were deemed graftable.

### Statistical Analysis

Descriptive statistics were calculated for demographic and clinical variables. Group comparisons used Student's *t* test (continuous) and χ^2^ tests (categorical). Univariate logistic regression identified significant predictors (p < 0.05), which were included in multivariate models. Stepwise logistic regression developed parsimonious models maximizing predictive power. Diagnostic performance was evaluated using sensitivity, specificity, and accuracy. Receiver operating characteristic curves and area under the receiver operating characteristic curve (AUCs) compared discriminative ability between modalities. Separate analyses were performed for the entire cohort and injury subgroups using SPSS version 28.0 (IBM Corp), with p < 0.05 considered significant.

## Results

### Patient Demographics

Of 841 patients with brachial plexus reconstruction, 439 were excluded: chronic injuries >12 months (n = 54), infraclavicular injuries (n = 89), primary free functioning muscle transplantation (n = 41), compression/iatrogenic injuries (n = 45), tumor-related pathology (n = 56), lower plexus BPI with intact C5 root (n = 46), MRI performed at outside institutions (n = 56), and thoracic outlet syndrome (n = 52). This resulted in 402 patients meeting inclusion criteria. All were of Asian ethnicity and sustained injuries from motorcycle or motor vehicle accidents. Intraoperative exploration revealed graftable C5 spinal nerves in 195 patients (48.5%). Graftable vs. nongraftable C5 groups demonstrated comparable baseline characteristics (Table I). Injury patterns were similarly distributed between the avulsion and graftable groups, with panplexus injuries occurring in 39.6% vs. 43.1% and non-panplexus injuries in 60.4% vs. 56.9% (p = 0.543).

**TABLE I:** Patient Demographics

	C5 Nongraftable	C5 Graftable	p
No. of patients	207	195	—
Gender			0.123
Male (%)	183(88.4%)	162 (83.1%)	—
Female (%)	24 (11.6%)	33 (16.9%)	—
Mean age ± SD, yr	34.89 ± 27.271	32.28 ± 20.543	0.065
Mean BMI ± SD, kg/m^2^	24.63 ± 5.023	24.38 ± 4.705	0.987
Injury pattern			0.543
Panplexus	82 (39.6%)	84 (43.1%)	—
Non-panplexus	125 (60.4%)	111 (56.9%)	—

BMI = body mass index.

### Univariate Analysis of Diagnostic Predictors in the Overall Population

In the overall cohort, MRN was the strongest predictor of C5 graftability (odds ratio [OR] 9.171), followed by a positive Tinel's sign (OR 3.601), rhomboid strength ≥MRC 2/5 (OR 2.988), and levator scapulae strength ≥MRC 2/5 (OR 2.389; Table II). Deafferentation pain and EMG findings showed no predictive value.

**TABLE II:** Univariate Analysis of Diagnostic Predictors for C5 Nerve Graftability

Parameters	OR		Odds Ratio		OR	
	All patients (n = 402)	p	Panplexus (n = 166)	p	Nonpanplexus (n = 236)	p
Deafferentation pain	0.854 (0.441-1.654)	0.764	0.892 (0.283-2.814)	1.000	0.971 (0.439-2.148)	0.941
Tinel sign at neck	3.601 (3.601-2.284)	<0.001	4.837 (2.364-9.898)	<0.001	3.047 (1.677-5.536)	<0.001
MP of rhomboid ≥2	2.988 (1.617-5.523)	<0.001	2.046 (0.871-4.806)	0.1	4.566 (1.798-11.596)	<0.001
MP of levator Scapulae ≥2	2.389 (1.347-4.237)	0.003	1.374 (0.611-3.091)	0.442	4.264 (1.775-10.241)	<0.001
MRN	9.171 (5.795-14.514)	<0.001	15.864 (7.333-34.321)	<0.001	7.322 (3.955-13.557)	<0.001
EMG (paraspinal C5)	1.485 (0.406-5.426)	0.55	1.091 (0.066,17.972)	0.951	1.512 (0.343-6.661)	0.584
EMG (rhomboid)	1.31 (0.754-2.277)	0.339	1.487 (0.645-3.429)	0.352	1.197 (0.572-2.505)	0.633

EMG = electromyography classified as preserved (detectable recruitment) or denervated (absent or severely reduced recruitment), MP = muscle power, MRN = magnetic resonance neurography, and OR = odds ratio.

MRN demonstrated optimal diagnostic performance with 80.7% specificity and 74.9% accuracy (Youden index 0.494; Table III). Tinel's sign showed higher sensitivity (73.4%) but lower specificity (56.4%) and accuracy (64.7%). EMG had very low specificity (<20%) for both the C5 paraspinal muscle and rhomboid.

**TABLE III:** Sensitivity and Specificity of the Diagnostic Predictors Relative to Patterns of Injury

Parameters	Sensitivity % (95% CI)			Specificity% (95% CI)			Accuracy% (95% CI)			PPV% (95% CI)			NPV% (95% CI)		
	ALL	Panplexus	Non-panplexus	ALL	Panplexus	Non-panplexus	ALL	Panplexus	Non-panplexus	ALL	Panplexus	Non-panplexus	ALL	Panplexus	Non-panplexus
Deafferentation pain	86.1 (79.8-90.6)	88.4 (78.8-94.0)	84.3 (75.3-90.4)	12.7 (8.4-18.9)	7.8 (3.4-17.0)	16.1 (10.0-24.9)	49.5 (44.0-55.0)	49.6 (41.3-58.0)	49.5 (42.3-56.7)	49.8 (43.9-55.7)	50.8 (42.0-59.6)	49.0 (41.2-56.9)	47.6 (33.4-62.3)	38.5 (17.7-64.5)	51.7 (34.4-68.6)
Tinel sign at neck	73.5 (66.3-79.6	73.0 (61.9-81.8)	73.9 (64.1-81.8)	56.5 (49.1-63.6)	64.2 (52.2-74.6)	51.8 (42.6-60.9)	64.7 (59.5-69.6)	68.8 (60.7-75.9)	61.9 (55.0-68.3)	61.3 (54.4-67.8)	69.2 (58.3-78.4)	56.2 (47.3-64.7)	69.4 (61.5-76.4)	68.3 (56.0-78.4)	70.4 (59.7-79.2)
MP of Rhomboid ≥ 2	91.4 (86.4-94.6)	87.7 (78.7-93.2)	94.2 (88.0-97.3)	22.1 (16.8-28.4)	22.4 (14.5-32.9)	21.8 (15.4-30.1)	55.8 (50.8-60.7)	56.1 (48.2-63.6)	55.6 (49.0-62.0)	52.6 (47.2-58.0)	54.6 (46.0-62.9)	51.3 (44.3-58.3)	72.9 (60.4-82.6)	63.0 (44.2-78.5)	81.3 (64.7-91.1)
MP of Lev. Scapu. ≥ 2	89.1 (83.8-92.9)	83.8 (74.2-90.3)	93.3 (86.8-96.7)	22.6 (17.3-28.9)	21.1 (13.4-31.5)	23.5 (16.8-31.9)	54.9 (49.8-59.8)	53.2 (45.4-60.9)	56.1 (49.5-62.4)	52.1 (46.6-57.5)	52.8 (44.1-61.2)	51.6 (44.5-58.6)	68.8 (56.6-78.8)	55.2 (37.5-71.6)	80.0 (64.1-89.9)
MRN	68.7 (61.9-74.8)	84.5 (75.3-90.7)	56.8 (47.5-65.6)	80.7 (74.8-85.5)	74.4 (64.0-82.6)	84.8 (77.5-90.0)	74.9 (70.4-78.9)	79.5 (72.7-85.0)	71.6 (65.5-77.0)	77.0 (70.2-82.6)	77.2 (67.6-84.6)	76.8 (66.6-84.6)	73.2 (67.1-78.6)	82.4 (72.2-89.4)	68.8 (61.1-75.6)
EMG (paraspinal C5)	96.0 (90.3-98.4)	98.0 (89.3-99.6)	94.2 (84.4-98.0)	5.8 (2.7-12.0)	2.2(0.4-11.6)	8.5 (3.7-18.4)	50.2 (43.5-57.0)	52.1 (42.1-61.9)	48.6 (39.6-57.8)	49.7 (42.8-56.7)	52.2 (42.1-62.1)	47.6 (38.2-57.1)	60.0 (31.3-83.2)	50.0 (9.5-90.5)	62.5 (30.6-86.3)
EMG (Rhomboid)	85.2 (79.3-89.6)	85.2(75.9-91.3)	85.1 (76.9-90.8)	18.6 (13.6-24.8)	20.5 (12.9-31.2)	17.3 (11.3-25.4)	51.8 (46.7-56.9)	54.5 (46.7-62.2)	49.8 (43.1-56.5)	51.0 (45.4-56.6)	54.3 (45.7-62.7)	48.6 (41.3-55.9)	55.7 (43.3-67.5)	55.6 (37.3-72.4)	55.9 (39.5-71.1)

CI = confidence interval, EMG = electromyography classified as preserved (detectable recruitment) or denervated (absent or severely reduced recruitment), MP = muscle power, MRN = magnetic resonance neurography, NPV = negative predictive value, and PPV = positive predictive value.

### Univariate Analysis in Injury Pattern Subgroup

Panplexus injuries (n = 166) showed enhanced MRN performance (OR 15.864) and significant Tinel's sign prediction (OR 4.837), while MP assessments lost significance (Table II). Non-panplexus injuries (n = 236) demonstrated reduced MRN performance (OR 7.322) but enhanced prediction in the MP of rhomboid and levator scapulae, both demonstrating significant predictive value (Table II).

### Multivariate Predictive Models in Overall Population

The discriminative ability of individual and combined diagnostic tools was evaluated using multiple logistic regression models with AUC analysis (Supplementary Table 1). Among individual modalities, MRN demonstrated the highest diagnostic accuracy (AUC 0.747), while EMG showed the lowest performance. Combining physical examination with MRN achieved AUC 0.799, suggesting synergistic diagnostic value.

**Fig. 1:**
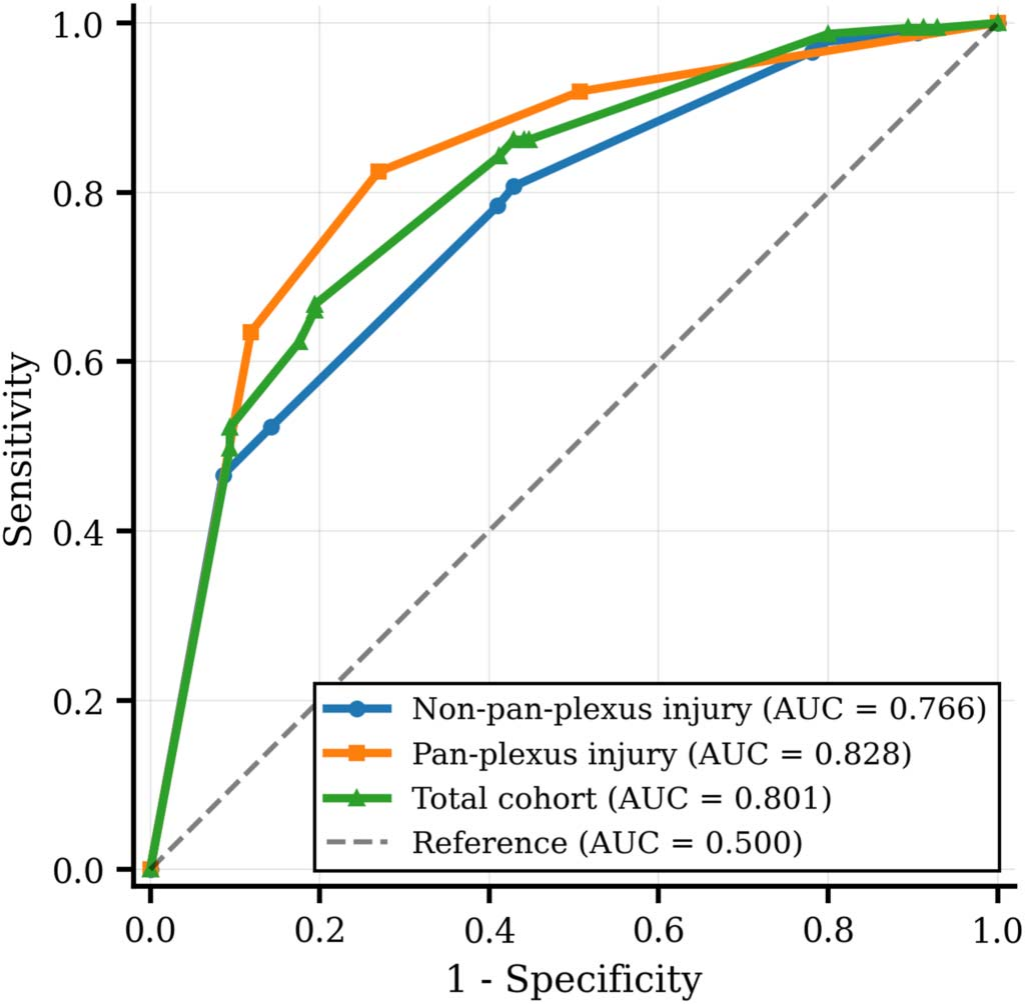
ROC Curves for Multivariable Parsimonious Model of C5 Nerve Graftability. ROC, receiver operating characteristic.

Assessment of multicollinearity using Variance Inflation Factors confirmed acceptable independence among all predictor variables (variance inflation factors range 1.060-2.740, all <5). Stepwise logistic regression identified an optimal three-variable model incorporating MRN (OR 6.140), Tinel's sign (OR 2.639), and rhomboid MP (OR 3.411), achieving AUC 0.801 (Fig. 1 and Table IV).

**TABLE IV:** Multivariable Parsimonious Model Based on Stepwise Selection High-Performance Logistic Regression

	ALL (n = 402)		Panplexus (n = 166)		Non-panplexus (n = 236)	
	OR of Graftable C5 Spinal Nerve	p	OR of Graftable C5 Spinal Nerve	p	OR of Graftable C5 Spinal Nerve	p
MRN	6.140 (3.516-10.723)	<0.001	10.333 (4.497-23.742)	<0.001	4.693 (2.332-9.444)	<0.001
Tinel sign	2.639 (1.503-4.635)	<0.001	3.294 (1.441, 7.532)	0.005	2.467 (1.246-4.885)	0.01
MP of rhomboid	3.411 (1.493-7.792)	0.004				
MP of levator scapulae					4.775 (1.63-13.99)	0.004
EMG paraspinal C5 + EMG rhomboid	2.464 (1.036-5.861)	0.041				
AUC	0.801		0.828		0.766	

AUC = area under the receiver operating characteristic curve, EMG = electromyography, MP = muscle power, MRN = magnetic resonance neurography, and OR = odds ratio

### Injury Pattern-Specific Models

Subgroup analysis using the same stepwise modeling approach revealed distinct optimal predictors for each injury pattern, maximizing predictive value with the fewest variables (Fig. 1 and Table IV). For panplexus injuries, the most effective model incorporated MRN (OR 10.333) and Tinel's sign (OR 3.294), achieving AUC 0.828. Non-panplexus injuries demonstrated a different profile, with the optimal model including MRN (OR 4.693), Tinel's sign (OR 2.467), levator scapulae MP (OR 4.775), yielding AUC 0.766.

## Discussion

Accurate preoperative assessment of C5 nerve root graftability remains one of the most challenging aspects of traumatic brachial plexus reconstruction, directly influencing surgical strategy and patient outcomes. This large cohort provided the statistical power for comprehensive injury-pattern subgroup analysis not feasible in previous investigations. Our systematic comparison revealed distinct diagnostic requirements for different injury patterns: Prediction of C5 graftability in panplexus injuries was optimized using MRN and Tinel’s sign, whereas non-panplexus injuries necessitated the inclusion of MP assessment. Furthermore, combining these modalities provided a quantifiable synergistic benefit, increasing the diagnostic accuracy (AUC 0.799-0.828) compared with MRN assessment alone (AUC 0.747).

### MRN’s Diagnostic Superiority and Implementation Barriers

MRN emerged as the most powerful diagnostic tool for C5 graftability assessment in this study. High-resolution FIESTA neurography provides direct visualization of intradural nerve rootlets, enabling differentiation between avulsion and rupture—anatomical information unattainable through other modalities^14^. Its high specificity (80.7% overall) ensures positive findings reliably guide surgical planning, minimizing false-positive assessments.

MRN proves particularly valuable in panplexus injuries where extensive damage limits clinical examination, especially when planning C5 grafting to the median nerve using free vascularized ulnar nerve flap. In cases confounded by comorbidities (clavicle fractures, vascular injuries), MRN provides objective anatomical information, preventing oversight of potentially graftable roots.

Despite demonstrated superiority, widespread MRN adoption faces barriers including institutional familiarity with traditional myelography and preference for intraoperative evoked potentials^16,17^. This perspective may reflect suboptimal protocols or poor image quality at some institutions. Our superior performance likely stems from optimized protocols specifically tailored for traumatic BPI. High-resolution 3D MR myelography with 0.6 to 0.8 mm^3^ isotropic voxels enables multiplanar reconstruction critical for root evaluation. Nerve-selective sequences with gadolinium further enhance diagnostic confidence by improving nerve-to-muscle contrast and branch nerve conspicuity^18^.

While computed tomography myelography has historically been considered the gold standard for detecting nerve root avulsions—with reported sensitivities ranging from 80% to 95%—it is a relatively more invasive procedure. MRN provides a noninvasive alternative with comparable diagnostic reliability in rootlet evaluation, while also offering improved assessment of extraforaminal nerve segments.

### Clinical Examination and Electrodiagnostic Assessment

Tinel's sign emerged as the only physical examination finding maintaining significance across both injury patterns, demonstrating 73.4% sensitivity and 56.4% specificity overall. Predictive value was enhanced in panplexus injuries (OR 4.837) versus non-panplexus (OR 3.047), likely because MRN provides objective anatomical assessment that is not affected by the extensive muscle denervation that limits reliability of clinical examination in panplexus injuries. These results align with previous reports: Addosooki et al.^19^ reported sensitivity of 77.8% and specificity of 55.6%, while Echalier et al.^20^ achieved sensitivity of 100% and specificity of 80% for detecting graftable C5 and/or C6 roots. Positive Tinel's sign correlates with regenerating axons, confirming viable proximal stumps suitable for grafting^21^, while its absence in complete avulsion reflects anatomical discontinuity precluding nerve reconstruction.

Our analysis revealed significant EMG limitations for C5 graftability assessment, with both C5 paraspinal and rhomboid EMG demonstrating limited predictive value^22^. Technical factors contribute to poor performance: High proportion of missing data introduces selection bias; needle misplacement (particularly for deep rhomboid beneath trapezius) represents significant error^23^; and signal crosstalk from adjacent muscles reduces specificity^24^. These findings argue against routine electrodiagnostic studies for C5 graftability prediction.

### Clinical Implications of MRN in Brachial Plexus Decision Making

Although surgical exploration remains definitive, integrating MRN into preoperative planning provides substantial strategic benefits. First, MRN aids intraoperative decision-making by helping prioritize reconstruction targets. When MRN confirms a high-quality ruptured C5, it is prioritized for critical targets; conversely, if MRN suggests poor quality despite intraoperative continuity, the C5 root may be relegated to lower-priority targets while reliable distal transfers secure essential functions. This “risk-stratified” approach maximizes C5 utility while safeguarding functional outcomes. Second, accurate assessment of C5 graftability preserves limited extraplexus donors. Identifying graftable spinal nerves allows surgeons to save critical donors, such as the spinal accessory nerve, for other reconstructions like free functioning muscle transplantation. To facilitate clinical application, we provide an MRN-based decision-making algorithm for C5 graftability (Supplementary Fig. 1).

### Study Limitations

Several limitations should be acknowledged. First, surgeons were not blinded to MRN findings when making intraoperative graftability determinations, reflecting real-world surgical planning where imaging guides approach. While this introduces a theoretical risk of confirmation bias, this was mitigated by the use of strict, objective intraoperative criteria—specifically the requirement for dual-surgeon consensus and physiological confirmation via dorsal scapular nerve stimulation—which provided a definitive reference standard independent of the surgeon's subjective impression. Second, retrospective design introduces potential selection bias, though large sample size mitigates this. Third, single-institution conduct with specific expertise may limit generalizability to centers with different protocols. Fourth, no concurrent intraoperative neuromonitoring was performed with MRN in the same patients, and thus, no comparative data were available. Finally, long-term functional outcomes were not assessed, limiting ability to correlate diagnostic accuracy with ultimate patient function.

## Conclusions

MRN is a valuable tool for assessing C5 nerve root graftability in traumatic BPI, demonstrating the highest predictive performance among evaluated modalities. When integrated with targeted clinical examination, MRN supports structured preoperative decision-making. While surgical exploration remains definitive, this multimodal approach may improve prioritization of reconstructive strategies and optimize use of limited donor nerves.

## Appendix

Supporting material provided by the authors is posted with the online version of this article as a data supplement at jbjs.org (http://links.lww.com/JBJSOA/B208, http://links.lww.com/JBJSOA/B209). This content was not copyedited or verified by JBJS.
